# Endometrial and vaginal microbiomes influence assisted reproductive technology outcomes

**DOI:** 10.5935/1518-0557.20220040

**Published:** 2023

**Authors:** Maho Miyagi, Keiko Mekaru, Suguru E. Tanaka, Wataru Arai, Kyota Ashikawa, Yoshiyuki Sakuraba, Rie Nakamura, Sugiko Oishi, Kozue Akamine, Yoichi Aoki

**Affiliations:** 1 Department of Obstetrics and Gynecology Graduate School of Medicine University of the Ryukyus Nishihara, Okinawa, Japan; 2 Varinos Incorporated, Aomi, Tokyo, Japan

**Keywords:** Endometrium, vagina, microbiota, *in vitro* fertilization, pregnancy, assisted reproductive technology

## Abstract

**Objective:**

The role of *Lactobacillus*-dominant microbiota in the endometrium in reproductive function is unclear. We therefore aimed to explore the impact of the balance of *Lactobacillus* and pathological bacteria in the endometrial and vaginal microbiomes on the pregnancy outcomes of women treated with assisted reproductive technology (ART).

**Methods:**

This study included 35 women with infertility submitted to good-quality embryo transfers. The cutoff values for abundance of *Lactobacillus* species (spp.) and pathological bacteria in the endometrium and vagina were calculated. Women with *Lactobacillus* spp. and pathological bacteria abundance above the cutoff values were categorized in the high-abundance group, whereas those with abundance below cutoff values were categorized in the low abundance group. We divided the patients into four groups based on the combination of high/low abundance of *Lactobacillus* spp. and pathological bacteria.

**Results:**

The 35 cases of good-quality embryo transfer resulted in 21 pregnancies. Pregnant women were present in significantly higher proportions in the high *Lactobacillus* spp. abundance and low pathological bacteria abundance group, whereas the opposite combination (i.e., low *Lactobacillus* spp. abundance and high pathological bacteria abundance) saw a significantly higher proportion of nonpregnant women (*p*=0.022).

**Conclusions:**

The balance between *Lactobacillus* and pathological bacterial abundance in the endometrial and vaginal microbiomes is associated with pregnancy from ART.

## INTRODUCTION

Bacterial vaginosis (BV) is a vaginal infection marked by the reduction of vaginal *Lactobacillus* and proliferation of anaerobic bacteria, such as *Gardnerella vaginalis*, which affects 19% of patients with infertility (van Oostrum *et al*., 2013). The causative bacteria of BV include *G. vaginalis* and *Atopobium vaginae*. The pregnancy rate achieved using *in vitro* fertilization (IVF) was reported to be significantly lower in women with abnormal vaginal microbiota with high levels of bacterial abundance (Haahr *et al*., 2016). However, a meta-analysis showed that BV and abnormal vaginal microbiota had no effect on IVF pregnancy (Haahr *et al*., 2019). Nevertheless, with the advent of next-generation sequencing, a minute number of bacteria can now be comprehensively detected, and a paradigm shift has emerged in the understanding of the genital microbiome. The thorough detection of endometrial and vaginal microbiomes using next-generation sequencing may drastically impact the diagnosis of BV, which is typically assessed using microbial culture and quantitative PCR, and its relevance to IVF outcomes (Baker *et al*., 2018).

Moreno *et al.* (2016) defined *Lactobacillus*-dominant microbiota (LDM) as the group with ≥90% of *Lactobacillus* spp. in the endometrial microbiota, and non-*Lactobacillus-*dominant microbiota (NLDM) as that with <90% of *Lactobacillus* spp; consequently, *Lactobacillus* predominance was associated with successful implantation and lower miscarriage rates in assisted reproductive technology (ART). However, IVF pregnancy rates were not significantly different between the LDM and NLDM groups (Kyono *et al*., 2018a; Hashimoto & Kyono, 2019). Hashimoto & Kyono (2019) defined eubiosis and dysbiosis as *Lactobacillus* spp. + *Bifidobacterium* spp. at levels ≥80% and <80%, respectively. IVF pregnancy rates were not significantly different between these two groups.

Previous research has suggested that *Lactobacillus* abundance is crucial for implantation (Moreno *et al*., 2016; Koedooder *et al*., 2019). However, the effect of pathological bacteria on pregnancy outcomes of ART remains unclear, thereby raising the question whether the abundance of only *Lactobacillus* spp. or also of pathological bacteria is associated with ART pregnancy. We therefore hypothesized that a balance between the abundance of *Lactobacillus* and pathological bacteria influences pregnancy and thus aimed to explore the potential impact of this balance in the endometrial and vaginal microbiomes on ART pregnancy outcomes.

## MATERIALS AND METHODS

### Research Setting and Study Population

This prospective cohort study examined the endometrial and vaginal microbiomes of 35 patients who underwent good-quality embryo transfer using ART at the University of the Ryukyus’ Hospital between February 2019 and March 2020.

A good-quality embryo was defined as a blastocyst with a Gardner classification of 3BB or higher (Gardner *et al*., 2000). Endometrial and vaginal samples were obtained on days 8-10 of the menstrual cycle prior to transfer for 16S rRNA analysis using a next-generation sequencer. On day 15 of the hormone replacement cycle, endometrial thickness was measured by transvaginal ultrasonography, and a frozen embryo transfer was performed five days later. If endometrial thickness was at least 8 mm, transfer was possible; one or two good-quality blastocysts were then transferred.

Among the 35 cases, one vaginal and two endometrial samples were excluded from microbiome analysis because they had similar microbial communities as that of the negative control. Finally, 34 vaginal and 33 endometrial samples were analyzed. As a subanalysis, we excluded patients who were administered antibiotics (n=10) and examined 24 cases of good-quality embryo transfer. Among the 24 cases, one endometrial sample was excluded from microbiome analysis because it had a similar microbial community as that of the negative control. Finally, 24 vaginal and 23 endometrial samples were analyzed. Further, we divided the patients into four groups based on the combination of high/low abundance of *Lactobacillus* spp. and pathological bacteria as determined in the main analysis.

Thirty-three species of bacteria, including *Gardnerella* spp. and *Prevotella* spp., which cause BV and endometritis, were defined as pathological bacteria in this study (Table 1) (Moreno & Simon, 2018; Hillier *et al*., 1993; Funke *et al*., 1998; Scanziani *et al*., 1999; Cox & Slack, 2002; Haggerty *et al*., 2004; Polanco *et al*., 2012; Carlstein *et al*., 2016; Onderdonk *et al*., 2016; Petrina *et al*., 2017; Sweeney *et al*., 2016; Kaambo *et al*., 2018; Tao *et al*., 2019). The cutoff values of *Lactobacillus* spp. abundance and pathological bacteria abundance in the endometrium and vagina of all women were calculated using the receiver operating characteristic (ROC) curve. Women with *Lactobacillus* spp. abundance above and below the cutoff value were classified into the high-abundance *Lactobacillus* (High L) and low-abundance *Lactobacillus* (Low L) groups, respectively. Women with pathological bacteria abundance above and below the cutoff value were classified into the high-abundance pathological bacteria (High PB) and low-abundance pathological bacteria (Low PB) groups, respectively. These groups were further combined according to the balance of endometrial and vaginal microbiomes and used to categorize the patients into four groups, each examined for associations with pregnancy outcome.

**Table 1:** Pathological bacteria identified in female reproductive organs from past studies.

*Anaerococcus tetradius*
*Atopobium vaginae*
*Bacteroides caccae*
*Bacteroides dorei*
*Bacteroides fragilis*
*Bacteroides stercoris*
*Bacteroides uniformis*
*Bacteroides xylanisolvens*
*Enterococcus faecalis*
*Escherichia coli*
*Finegoldia magna*
*Fusobacterium nucleatum*
*Gardnerella vaginalis*
*Haemophilus parainfluenzae*
*Megasphaera (Unclassified)*
*Mobiluncus (Unclassified)*
*Mycoplasma hominis*
*Parabacteroides merdae*
*Peptostreptococcus anaerobius*
*Porphyromonas uenonis*
*Prevotella bivia*
*Prevotella buccalis*
*Prevotella corporis*
*Prevotella denticola*
*Prevotella disiens*
*Prevotella intermedia*
*Prevotella oris*
*Prevotella timonensis*
*Sneathia amnii*
*Streptococcus agalactiae*
*Streptococcus anginosus*
*Ureaplasma (Unclassified) (U. parvum)*
*Ureaplasma urealyticum*

Ravel *et al*. (2011) evaluated the vaginal microbiome according to the vaginal community state type (CST), which categorizes the genus *Lactobacillus* by species. CST is a classification of *Lactobacillus* species with high abundance in the vaginal microbiome. The vaginal microbiome was classified into CST I-CST V, where CST I, CST II, CST III, CST IV, and CST V was classified as *L. crispatus, L. gasseri, L. iners*, a diverse group dominated by species other than *Lactobacillus*, and *L. jensenii* (Ravel *et al*., 2011).

The primary endpoint was the association of endometrial and vaginal microbiomes with pregnancy outcomes.

This study conformed to the principles of the Declaration of Helsinki (revised in October 2013) and the Ethical Guidelines for Medical Research Involving Human Subjects (Ministry of Education, Culture, Sports, Science and Technology and Ministry of Health, Labour, and Welfare Notification No. 3, 2014). Written consent was obtained from all eligible patients. Additionally, the University of the Ryukyus’ Medical Research Ethics Review Committee for Human Subjects approved this research (Approval No. 1354).

### ART

ART included conventional IVF, intracytoplasmic sperm injection (ICSI), and frozen-thawed embryo transfer. During egg retrieval, ovarian stimulation was performed using the short method or the antagonist method as a controlled ovarian stimulation method in patients without ovarian hypofunction. Additionally, patients were administered 225-300 IU/day of human menopausal gonadotropin. For patients with low ovarian function, mild-stimulation methods, such as clomiphene and natural cycles, were used. When two or more follicles with a diameter ≥18 mm were found, oocyte retrieval surgery was performed. On the same day, ovulation was induced with 5000 units of human chorionic gonadotropin intramuscular injection, followed by oocyte retrieval surgery after 36 h. The embryos obtained by IVF or ICSI were frozen as blastocysts, and hysteroscopy and sample collection were performed in the cycle before thawed embryo transfer.

In cases of hysteroscopy with suspicious findings of chronic endometritis, such as strawberry redness, localized congestion, bleeding points, micropolyps, and interstitial edema (Cicinelli *et al*., 2019), two antibiotics, namely levofloxacin (500 mg/day for 7 days) and cefotiam (600 mg/day for 7 days), were administered. In frozen-thawed embryo transfers, one or two good-quality blastocysts were transferred under hormone replacement cycle. Hormone replacement therapy was initiated with estrogen preparations on days 2-5 of menstruation; when an endometrial thickness >8 mm was achieved on day 15, luteal hormone preparation was initiated. After five days of luteal hormone preparation, the blastocyst was transferred. In case of successful pregnancy, hormone replacement therapy was continued until nine weeks of gestation.

### Sample Collection

On days 8-10 of the menstrual cycle before embryo transfer, vaginal and endometrial secretion specimens were obtained simultaneously during hysteroscopy.

Before vaginal disinfection, vaginal secretions were collected with a swab. The vagina was then disinfected thrice with benzalkonium chloride solution and rinsed with saline solution. Finally, endometrial secretions were collected transvaginally with a cell-collecting brush (YuinoBrush^®^, Asuka Pharmaceuticals, Tokyo, Japan).

### Bacterial Species Analysis

Bacterial species were identified by amplifying the variable regions 1-2 (V1-V2) of the 16S rRNA gene and using next-generation sequencing.

### DNA Extraction, Sequencing, and Analysis

DNA was extracted from the endometrial and vaginal secretions of pregnant and nonpregnant patients using a previously reported protocol (Kyono *et al*., 2018b; Mariya *et al*., 2021). UltraPure™ DNase/RNase-Free Distilled Water (Thermo Fisher Scientific Inc., Waltham, MA, USA) was used as a negative control. After amplifying the V1-V2 regions of the bacterial 16S rRNA gene, the final library was paired-end-sequenced at 2 × 251 bp using a MiSeq Reagent Kit v3 on an Illumina MiSeq platform (Illumina, Inc., San Diego, CA, USA). After quality filtering of the paired-end reads, operational taxonomic units (OTUs) were created. The OTUs were then assigned to taxonomy using a database used in a previous study (Mariya *et al*., 2021). Bacteria frequently observed in the negative control were considered to be background bacterial contamination (Table 2); these bacteria were excluded from the microbiome profile after sample screening.

**Table 2:** Bacteria excluded from the analysis as they were frequently observed in the negative control.

*Acidovorax delafieldii*	*Afipia broomeae*	*Pandoraea apista*	*Ralstonia pickettii*
*Acinetobacter bereziniae*	*Brevundimonas diminuta*	*Phyllobacterium myrsinacearum*	*Serratia marcescens*
*Acinetobacter guillouiae*	*Cupriavidus metallidurans*	*Pseudomonas extremorientalis*	*Stenotrophomonas maltophilia*
*Aeromonas salmonicida*	*Delftia lacustris*	*Pseudomonas migulae*	*Stenotrophomonas pavanii*
*Afipia birgiae*	*Delftia tsuruhatensis*	*Pseudomonas rhodesiae*	*Vibrio metschnikovii*

### Sample Screening and Statistical Analysis

Non-hierarchical clustering of microbiome profiles in endometrial secretions, vaginal secretions, and negative control was performed using the weighted Unifrac distance. Samples clustered as negative controls were excluded from the following analysis. Next, non-hierarchical clustering of microbiome profiles in endometrial and vaginal secretions, excluding background contaminant bacteria, was performed using the weighted Unifrac distance and Gap statistics on the principal coordinate analysis (PCoA) plot. We also investigated the bacterial contribution to the ordination biplot of PCoA. Finally, hierarchical clustering of endometrial and vaginal secretions with microbiome profiles, excluding background contaminant bacteria, was performed using the Bray-Curtis distance. Subsequently, heat maps were generated.

All analyses were performed using R software version 3.6.2. The normality and the homoscedasticity of continuous data were analyzed using the Shapiro-Wilk and Bartlett’s tests, respectively. When the data were both normally distributed and homoscedastic, Student’s *t*-test was used, whereas when they were only normally distributed but not homoscedastic, Welch’s *t*-test was used. For non-normally distributed data, the Wilcoxon rank-sum test was used. For discrete data, Fisher’s exact test was used. Permutational multivariate analysis of variance (PERMANOVA) test was used in diversity and rarefaction analyses. A *p*-value of <0.05 was considered statistically significant.

## RESULTS

### Clinical Background and ART Results

Twenty-one of the 34 good-quality embryo transfer ended in pregnancy. Of these, 17 resulted in live births and four in miscarriages at 6-8 weeks. Table 3 summarizes the profiles of 21 pregnant and 13 nonpregnant women. Mean age was 35.6 years in pregnant women and 36.6 years in nonpregnant women (*p*=0.45), and their anti-Müllerian hormone levels were 3.53 and 3.14 ng/mL (*p*=0.69), respectively. The number of pregnancies, body mass index, infertility duration, and causes of infertility were not significantly different between the two groups. Hysteroscopy was immediately performed after microbiome collection. There were no patients with submucous polyps or fibroids. Ten patients with suspected chronic endometritis were treated with levofloxacin and cefotiam. Five became pregnant and five did not; pregnancy rates were not significantly different between patients given and patients not given antibiotics (Table 3).

**Table 3:** Comparison of clinical backgrounds and ART outcomes between pregnant and nonpregnant patients.

	Pregnant (n=21)	Nonpregnant (n=13)	*P*
Age (y) mean (SD) (95% CI) median (range)	35.6 (±0.77) (34.1-37.1) 36 (29-41)	36.6 (±0.98) (34.2-39) 36 (32-43)	0.45
Previous pregnancies mean (SD) (95% CI) median (range)	0.95 (±0.23) (0.47-1.43) 1 (0-3)	1.08 (±0.31) (0.45-1.71) 1 (0-4)	0.74
AMH (ng/mL) mean (SD) (95% CI) median (range)	3.53 (±0.61) (2.28-4.78) 2.79 (0.29-11.2)	3.14 (±0.77) (1.55-4.72) 2.12 (0.16-9.22)	0.69
BMI mean (SD) (95% CI) median (range)	22.7 (±0.8) (21-24.3) 22.5 (18.9-35.3)	22.2 (±1.02) (20.2-24.5) 21.9 (17.1-28.2)	0.78
Duration of infertility (y) mean (SD) (95% CI) median (range)	3.38 (±0.74) (1.86-4.90) 2 (0.5-13)	3.58 (±0.98) (1.57-5.59) 2 (1-12)	0.87
Infertility factor (n) Fallopian tube factor Male factor Endometriosis factor Diminished ovarian reserve^*^	5/21 (23.8%) 10/21 (47.6%) 5/21 (23.8%) 7/21 (33.3%)	3/13 (23%) 11/13 (84.6%) 3/13 (23%) 6/13 (46.1%)	1.00 0.06 1.00 0.49
Use of antibiotics (n)	5/21 (23.8%)	5/13 (38.5%)	0.45
Endometrial thickness at embryo transfer (mm) mean (SD) (95%CI) median (range)	10.2 (±0.35) 10 (8-14)	10.6 (±0.44) 10 (8-13.1)	0.48
No. of embryos transferred (n) mean (SD) (95%CI) median (range)	1.04 (±0.05) (0.94-1.15) 1 (1-2)	1.07 (±0.06) (0.94-1.21) 1 (1-2)	0.73

The clinical background and the number of embryos transferred or the thickness of the endometrium were not significantly different. AMH, anti-Müllerian hormone; ART, assisted reproductive technology; BMI, body mass index; CI, confidence interval; SD, standard deviation

* Diminished ovarian reserve; AMH < 1 ng/mL

### Endometrial and Vaginal Microbiomes and Pregnancy

**Figure 1:**
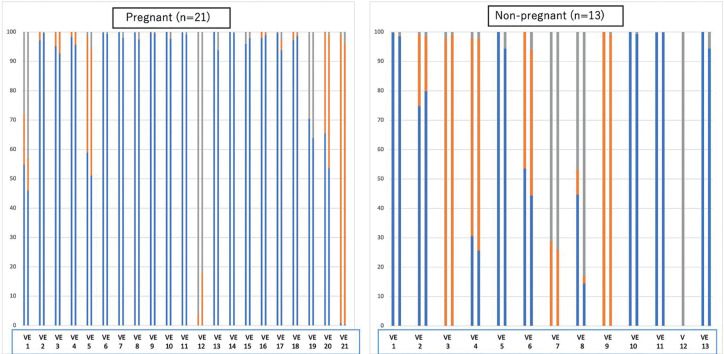
Abundance of *Lactobacillus* spp. and pathological bacteria in pregnant and nonpregnant women. Blue: Lactobacillus, Orange: Pathological bacteria, Gray: Other Vaginal/endometrial microbiome (vaginal: V, endometrial: E) per case in two adjacent bars. Pregnant patient numbers 1,7,9,12,15 and nonpregnant patient numbers 1,3,7,8,11 were diagnosed with chronic endometritis by hysteroscopy and treated with antibiotics. Several pregnant women had a high abundance of *Lactobacillus* spp. in both the endometrium and vagina, and many nonpregnant women had a high abundance of pathological bacteria.

Figure 1 illustrates the abundance of *Lactobacillus* spp. and pathological bacteria in pregnant and nonpregnant women. A high abundance of *Lactobacillus* spp. in both the endometrium and vagina was found primarily in pregnant women, whereas a high abundance of pathological bacteria was mainly found in nonpregnant women.

Additionally, excluding some cases, a strong correlation was found between the types of vaginal and endometrial microbiome (Figure 1).

### Cutoff Values for *Lactobacillus* spp. and Pathological Bacteria Abundance in the Endometrium and Vagina

As mentioned in the Materials and Methods section, ROC curves were used to establish the cutoff values for *Lactobacillus* spp. and pathological bacteria abundance in the endometrial and vaginal microbiomes of pregnant and nonpregnant women. The cutoff values for endometrial and vaginal *Lactobacillus* spp. abundance were 46% and 54.9%, respectively, whereas those for endometrial and vaginal pathological bacteria abundance were 18.7% and 8.5%, respectively. Fisher’s exact test was performed for endometrial and vaginal *Lactobacillus* spp. abundance for pregnancy outcomes using the cutoff values, and they indicated significant differences (endometrial microbiome: *p*=0.015, vaginal microbiome: *p*=0.007). Similarly, pathological bacteria abundance for pregnancy outcomes also indicated significant differences (endometrial microbiome: *p*=0.036, vaginal microbiome: *p*=0.042). Based on the balance of the endometrial and vaginal microbiomes, the patients were divided into the following four groups: (1) High L + Low PB, (2) High L + High PB, (3) Low L + Low PB, and (4) Low L + High PB.

### Relation between Endometrial Microbiome Balance and Pregnancy Outcomes

**Figure 2:**
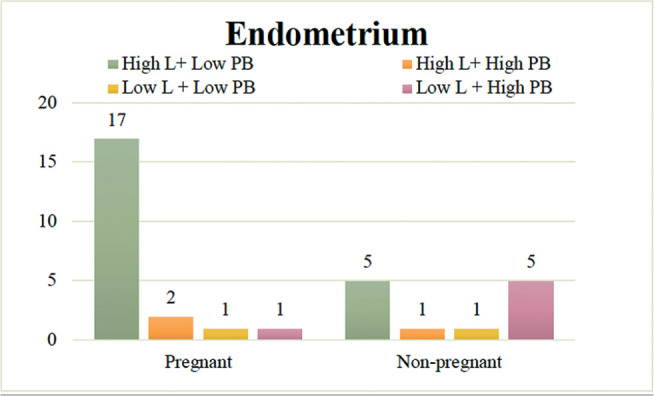
Pregnancy outcomes grouped according to the balance of *Lactobacillus* abundance and pathological bacteria abundance in the endometrium (n=33). The proportion of pregnant women was significantly higher (17/22: 77.3%) in High L + Low PB endometrial microbiomes, whereas the proportion of nonpregnant women was significantly higher (5/6: 83.3%) in Low L + High PB endometrial microbiomes (*p*=0.022) (Fisher’s exact probability test, one-tailed test). *High L: Group with high abundance of *Lactobacillus*, Low L: Group with low abundance of *Lactobacillus*, High PB: Group with high abundance of pathological bacteria, Low PB: Group with low abundance of pathological bacteria.

We found 22, 3, 2, and 6 cases of endometrial High L + Low PB, High L + High PB, Low L + Low PB, and Low L + High PB, respectively (Figure 2). Fisher’s exact test revealed that the pregnancies were significantly more frequent in the High L + Low PB endometrial microbiome group (17/22; 77.3%), whereas nonpregnant women were significantly more present in the Low L + High PB endometrial microbiome group (5/6; 83.3%) (*p*=0.022).

### Relation between Vaginal Microbiome Balance and Pregnancy Outcomes

**Figure 3:**
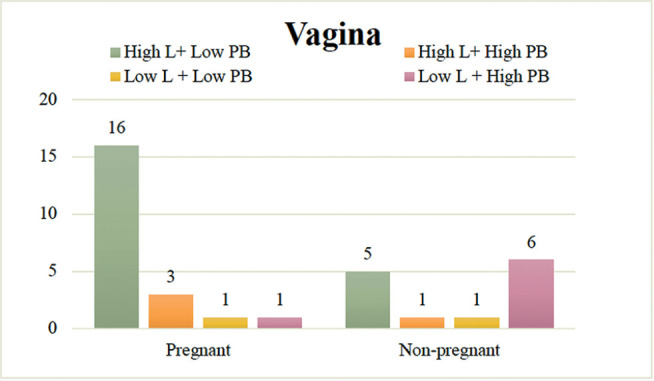
Pregnancy outcomes grouped according to the balance of *Lactobacillus* abundance and pathological bacteria abundance in the vagina (n=34). The proportion of pregnant women was significantly higher (16/21: 76.1%) in High L+ Low PB vaginal microbiomes, whereas the proportion of nonpregnant women was significantly higher (6/7: 85.7%) in Low L + High PB vaginal microbiomes (*p*=0.015) (Fisher’s exact probability test, one-tailed test). *High L: Group with high abundance of *Lactobacillus*, Low L: Group with low abundance of *Lactobacillus*, High PB: Group with high abundance of pathological bacteria, Low PB: Group with low abundance of pathological bacteria.

We found 21, 4, 2, and 7 cases of High L + Low PB, High L + High PB, Low L + Low PB, and Low L + High PB, respectively (Figure 3). Fisher’s exact test showed that pregnancies were significantly more frequent in the High L + Low PB vaginal microbiome groups (16/21; 76.2%), whereas nonpregnant women were significantly more present in the Low L + High PB vaginal microbiome groups (6/7; 85.7%) (*p*=0.015).

These results showed that the proportion of pregnant women was significantly higher in the High L + Low PB endometrial and vaginal microbiome groups, whereas proportion of nonpregnant women was significantly higher in the Low L + High PB endometrial and vaginal microbiome groups. Thus, the balance of *Lactobacillus* and pathological bacteria in the vaginal and endometrial microbiomes is related to pregnancy outcomes.

### Subanalysis: Excluding Patients Who Used Antibiotics

**Figure 4:**
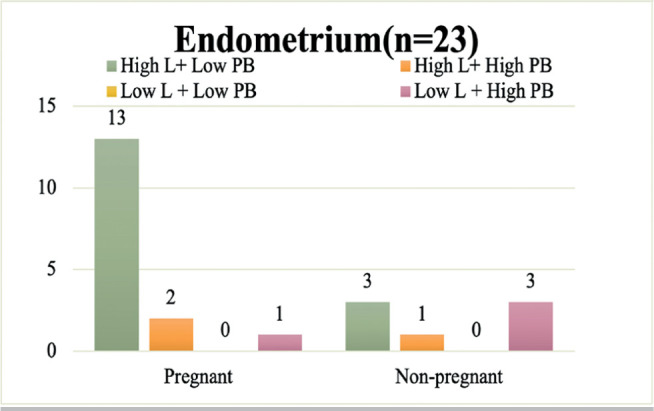
Pregnancy outcomes grouped according to the balance of *Lactobacillus* abundance and pathological bacteria abundance in the endometrium of patients not given antibiotics (n=23). In patients not given antibiotics (n=23), a trend (*p*=0.057) toward greater proportions of pregnant women (13/16: 81.2%) in the High L + Low PB group was observed, whereas a trend (*p*=0.057) towards greater proportions of nonpregnant women (3/4: 75%) in the Low L+ High PB group (*p*=0.057) was observed (Fisher’s exact probability test, one-tailed test). *High L: Group with high abundance of *Lactobacillus*, Low L: Group with low abundance of *Lactobacillus*, High PB: Group with high abundance of pathological bacteria, Low PB: Group with low abundance of pathological bacteria.

**Figure 5:**
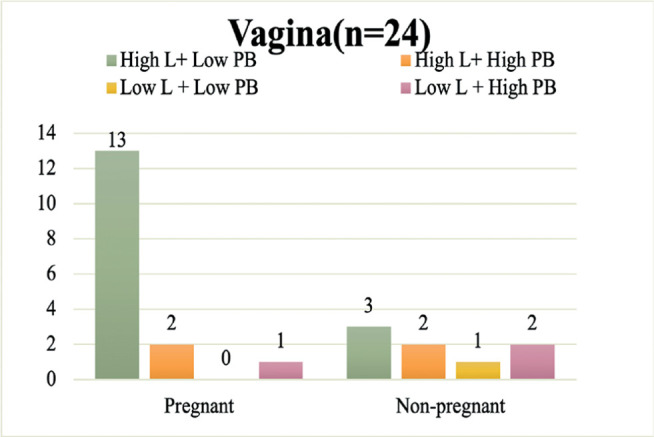
Pregnancy outcomes grouped according to the balance of Lactobacillus abundance and pathological bacteria abundance in the vagina in patients not given antibiotics (n=24). In patients not given antibiotics, the proportion of pregnant women was significantly higher (13/16: 81.2%) in the High L+ Low PB vaginal microbiomes, whereas the rate of nonpregnant women significantly higher (2/3: 66.7%) in the Low L+ High PB vaginal microbiomes (p=0.045) (Fisher’s exact probability test, one-tailed test). *High L: High abundance of the *Lactobacillus* group, Low L: Low abundance of the *Lactobacillus* group, High PB: High abundance of the pathological bacteria group, Low PB: Low abundance of the pathological bacteria group.

In subanalysis, we excluded patients given antibiotics (n=10) and examined 24 cases of good-quality embryo transfer. Sixteen of these cases ended in pregnancy. Additionally, there were no significant differences in patient clinical background between pregnant and nonpregnant women. We also divided the patients into four groups based on the combination of high/low abundance of *Lactobacillus* spp. and pathological bacteria, as determined in the main analysis. Further, one of the 24 endometrial samples was excluded from microbiome analysis because it had a similar microbial community as that of the negative control. In the 23 endometrial samples analyzed, higher proportions of pregnant women were observed in the High L + Low PB group, whereas nonpregnant women clustered around the opposite combination (Low L + High PB group) (*p*=0.057). Similarly, in the vaginal microbiome, greater proportions of pregnant women were observed in the High L + Low PB group, whereas higher proportions of nonpregnant women were observed in the opposite combination (Low L + High PB goup) (*p*=0.045). These results are shown in Figures 4 and 5. Thus, even after excluding patients given antibiotics, the balance between *Lactobacillus* and pathological bacteria abundance in the endometrial and vaginal microbiomes was associated with pregnancy outcomes from ART.

### Association between Vaginal Microbiome and Pregnancy According to CST

**Figure 6:**
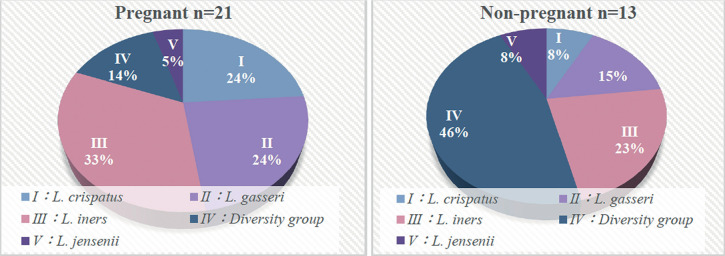
Community state type classification in pregnant and nonpregnant women. CST III (*Lactobacillus iners* dominant) tended to be more prevalent in pregnant women, whereas CST IV (diversity group) tended to be more prevalent in nonpregnant women. CST, community state type.

The breakdown of vaginal CST in both pregnant (n=21) and nonpregnant (n=13) groups showed that CST III (*L. iners* dominant) was the most common among pregnant women at 33%, followed by CST II (*L. gasseri* dominant) at 24%, CST I (*L. crispatus* dominant) at 24%, CST IV (diversity group) at 14%, and CST V (*L. jensenii* dominant) at 5%. In nonpregnant women, CST IV accounted for 46%, followed by CST III (23%), CST II (15%), CST I (8%), and CST V (8%). CST III was more common in pregnant women, whereas CST IV was more common in nonpregnant women (Figure 6). Although the association between CST classification and pregnancy or nonpregnancy was not statistically significant, CST IV tended to be more common in nonpregnant women (*p*=0.06).

### *β-diversity* Analysis

In the β-diversity analysis of the endometrial and vaginal microbiomes for pregnancy outcome, we used the *K*-means method to divide the cases into several clusters based on bacterial community, which were arranged using the Biplot method (Figures 7A, 8A) and PERMANOVA test (Figures 7B, 8B). Both endometrial and vaginal pregnancy rates were significantly higher in cases of high *Lactobacillus* spp. abundance and significantly lower in cases of high *Gardnerella* spp. abundance (Figures 7, 8) (PERMANOVA test for endometrium and vagina; *p*=0.048 and *p=*0.041, respectively).

**Figure 7a:**
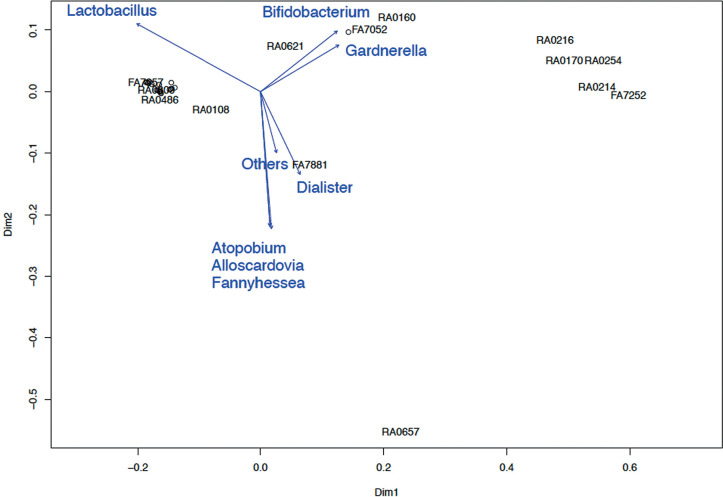
Trends in the types of endometrial bacteria based on the cluster indicated by the Biplot method. A high *Gardnerella* spp. abundance was associated with low pregnancy rates, whereas a high *Lactobacillus* abundance was associated with high pregnancy rates.

**Figure 7b:**
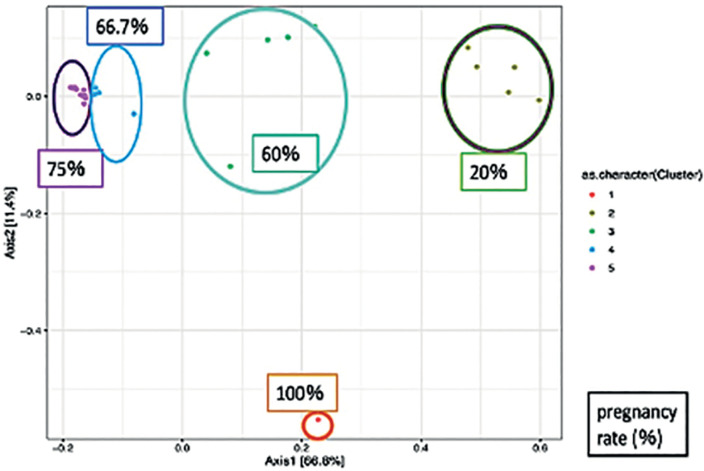
PERMANOVA test of trends in the type of endometrial bacteria and pregnancy rate using cluster (PCoA plot). In the biplot and PCoA plots, each plot is identical. The percentages shown in the figures are pregnancy rates. A high *Gardnerella* spp. abundance resulted in a low pregnancy rate, whereas a high *Lactobacillus* abundance resulted in a high pregnancy rate (PERMANOVA test *p*=0.048).

**Figure 8a:**
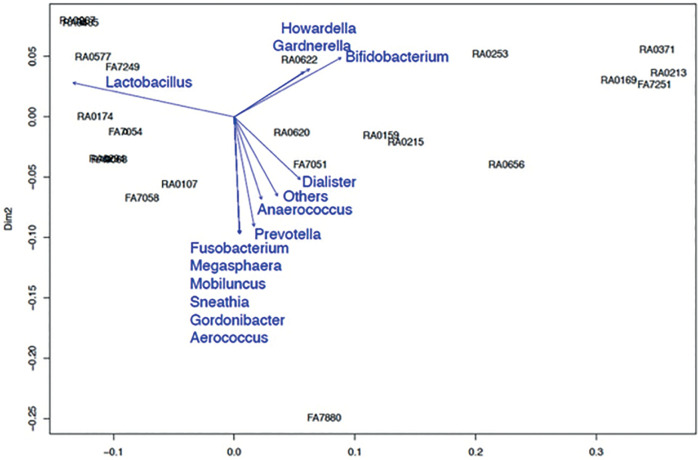
Trends in the types of vaginal bacteria based on the cluster indicated by the Biplot method. A high *Gardnerella* spp. abundance was associated with a low pregnancy rate, whereas a high *Lactobacillus* spp. abundance was associated with a high pregnancy rate.

**Figure 8b:**
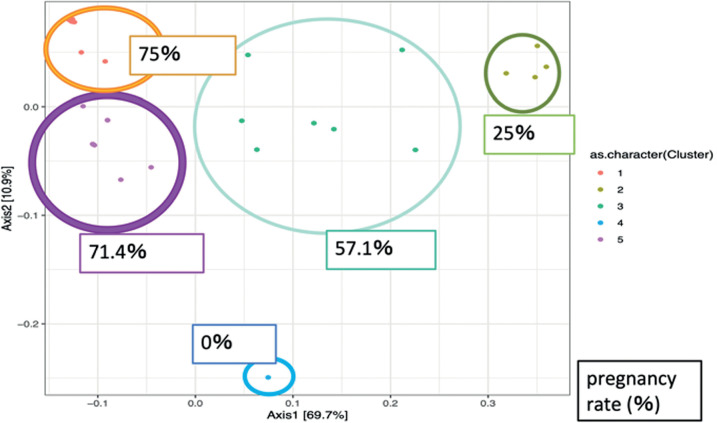
PERMANOVA test of trends in the type of vaginal bacteria and pregnancy rate using cluster (PCoA plot). In the biplot and PCoA plots, each plot is identical. The percentages shown in the figures are pregnancy rates. A high *Gardnerella* spp. abundance resulted in a low pregnancy rate, whereas a high *Lactobacillus* spp. abundance resulted in a high pregnancy rate (PERMANOVA test *p*=0.041).

These trends are also observed in the heat maps (Figure 9A,B). The heat map of the vaginal and endometrial microbiomes at a genus level showed that *Gardnerella* and other pathological bacteria were more common in nonpregnant patients, whereas *Lactobacillus* was more common in pregnant patients.

**Figure 9a:**
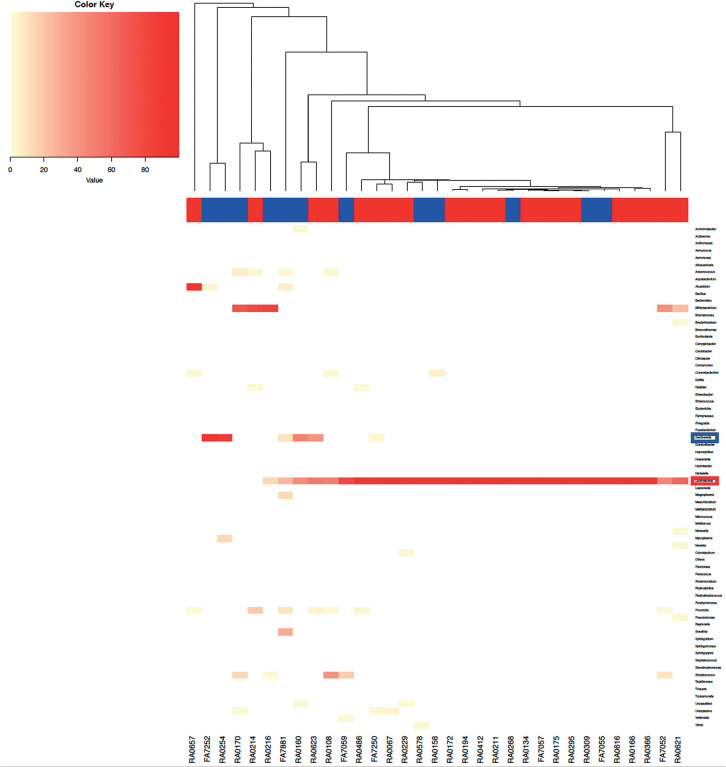
Heat map of the endometrial microbiome by genus. Pregnant and nonpregnant women are marked in red and blue, respectively. *Lactobacillus* and *Gardnerella* are circled in red and blue, respectively. *Lactobacillus* spp. is more common in pregnant women, whereas other bacteria are more common in nonpregnant women.

**Figure 9b:**
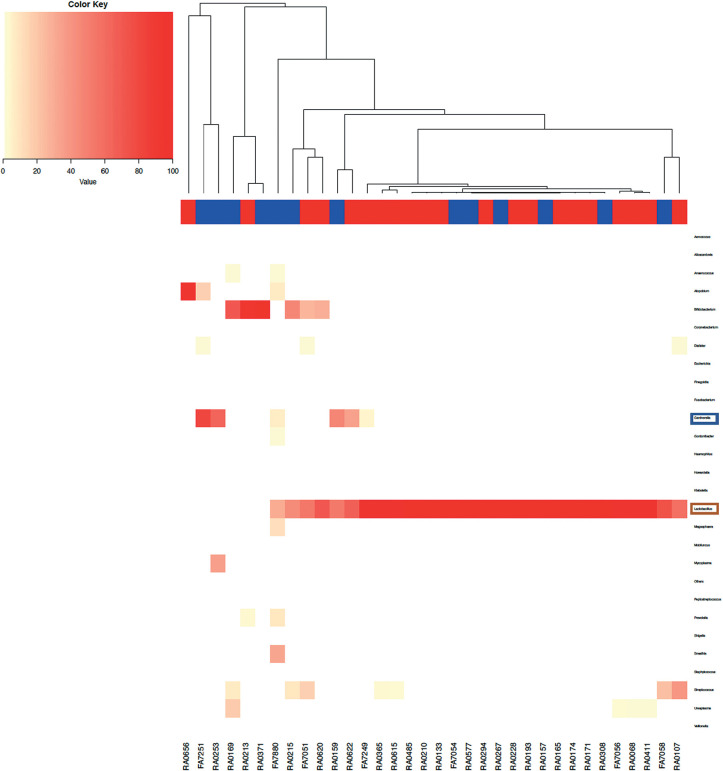
Heat map of the vaginal microbiome by genus. Pregnant and nonpregnant women are marked in red and blue, respectively. *Lactobacillus* and *Gardnerella* are circled in red and blue, respectively. Similar to the endometrial microbiome, Lactobacillus spp. is more common in pregnant women, whereas other bacteria (e.g., *Gardnerella*) are more common in nonpregnant women.

## DISCUSSION

This prospective study investigated the relationship between endometrial/vaginal microbiome and pregnancy in patients with infertility submitted to good-quality embryo transfers using ART. In both endometrial and vaginal samples, we found that significantly more women became pregnant when *Lactobacillus* and pathological bacteria abundance were high and low, respectively, and significantly more women were not pregnant when *Lactobacillus* and pathological bacteria abundance were low and high, respectively. Although many studies have focused on the high abundance of *Lactobacillus*, our study indicates that the balance between *Lactobacillus* and pathological bacteria may impact pregnancy outcomes.

### Validity of the Collection Method

This study is a prospective cohort study with an eligible clinical background. Thorough vaginal disinfection and cleaning were performed during sample collection to prevent contamination. To exclude background bacteria from the results, we established a negative control.

The endometrium is a low-biomass environment, with only 1/1000 of the bacteria present in the vagina (Moreno & Simon, 2018; Chen *et al*., 2017). Winters *et al*. (2019) obtained endometrial samples from an excised uterus. They reported that the endometrium was mainly composed of *Acinetobacter, Pseudomonas, Cloacibacterium*, and *Comamonadaceae*, contrary to the present findings and those of a previous study (Winters *et al*., 2019). This discrepancy could be attributed to the patients’ age. Their patients’ median age was 45 years and they had uterine fibroids and endometrial hyperplasia, whereas our patients’ median age was 36 years. A previous study suggests that the genital tract microbiome varies according to age and hormonal milieu (Moreno & Franasiak, 2017). Those authors also mentioned the possibility of the host’s DNA contaminating the swab samples during sample collection as a limitation of their study.

Additionally, Zervomanolakis *et al*. (2007) using hysterosalpingoscintigraphy to show that the uterus acts as a functionally peristaltic pump under the endocrine control of the ovary. They showed that 10-12 MBq ^99 m^Tc-radiolabeled microspheres on the cervix were taken up into the uterus within a few minutes during the follicular and luteal phases. Moreover, a previous study suggested that the endometrial microbiome is vaginal in origin (Baker *et al*., 2018). Therefore, the endometrial microbiome may correlate with the vaginal microbiome since it is influenced by microbial dissemination from the vagina. Even if vaginal contamination is considered, the composition of endometrial and vaginal paired samples is not identical, and bacterial composition has been reported to be significantly different between vaginal and uterine samples in approximately 20% of the patients (Moreno *et al*., 2016; Moreno & Simon, 2018). Therefore, sample collection via the transvaginal route with adequate precautions is clinically acceptable in clarifying the endometrial microbiome’s composition and characteristics.

### Link between the Endometrial/Vaginal Microbiomes and Pregnancy

The association between *Lactobacillus* predominance and good implantation rates is remarkable; however, the cutoff value for *Lactobacillus* abundance remains unclear. Additionally, *Gardnerella, Enterococcus*, Enterobacteriaceae, *Streptococcus*, and *Staphylococcus* are the causative bacteria of poor infertility outcome (Liu *et al*., 2019; Cicinelli *et al*., 2014; Salim *et al*., 2002; Selman *et al*., 2007). Therefore, this study defined them as a group of pathological bacteria. When we examined the relationship between pregnancy and pathological bacteria in terms of pathological bacteria abundance at the cutoff value based on the ROC curve, a high abundance of pathological bacteria was significantly more frequent in nonpregnant women than in pregnant women.

Table 4 shows the literature on the endometrial and vaginal microbiomes and ART outcomes. Generally, the number of cases per report is small, ranging from 10 to 190, and the method for measuring the microbiome varies in each study. Additionally, few comparisons were conducted with various clinical backgrounds, including age and embryo quality. The results were also presented mainly for *Lactobacillus* and *Gardnerella*, but none have focused on the balance in the abundance of BV causative agents, as in this study. Of the four studies focusing on the vaginal microbiome, two found that *Lactobacillus* predominance was associated with pregnancy by ART; one showed a non-significant trend in that same direction, and the other showed no association. Of the four studies on the endometrial microbiome, one found an association between *Lactobacillus* dominance and pregnancy with ART, another found a non-significant trend, and the others found no association. Therefore, the impact of the endometrial and vaginal microbiomes on ART pregnancy remains unclear. Further studies with a large number of patients are needed to establish a consistent patient background, measurement methods, and cutoff values.

**Table 4:** Articles on vaginal and endometrial microbiomes and ART outcomes (without repeated implantation failure).

Author	Sample (n)	Average age (y)	Analysis	Design	Relationship between microbiome and ART outcomes	ART outcomes (Pregnant, bioprofile)
**VAGINA**						
Hyman *et al*., 2012	30	38.5	BigDye Terminator, ABI 3730	Microbiome occupancy was assessed in patients who underwent IVF-ET.	not related	*Lactobacillus* is favorable but not sufficient for successful ET.
Moreno et al., 2016	13	39.5	454 pyrosequencing V3-V5	Pregnancy outcomes were compared in women who underwent IVF.	Related	NLDM (<90%) was associated with significant decreases in pregnancy (70.6% *vs*. 33.3%; *p*=.03) and live birth (58.8% *vs*. 6.7%; *p*=.002) rates.
Koedooder *et al*., 2019	192	31.5	Interspace profiling (IS-pro) molecular technique	Pregnancy outcomes were compared in 192 women who underwent IVF via fresh ET.	Related	High Lactobacillus abundance seemed to be related to IVF and ICSI. Women who had <60% *L. crispatus* had a high chance of pregnancy.
Bernabeu *et al*., 2019	31	40	V3-V4 Illumina MiSeq	Pregnancy outcomes were compared between 17 pregnant women and 14 nonpregnant women after undergoing ICSI-ET	tend to be related	α-diversity was found in nonpregnant patients compared with that in pregnant patients, although this difference was not significant (*p*=0.088).
Present study	35	36	Illumina MiSeq V1, V2	The vaginal microbiome profiles of 34 women who underwent good ET with IVF were compared (21 pregnant cases vs. 13 nonpregnant cases).	Related	The vaginal microbiomes of pregnant cases demonstrated significantly more cases of high abundance of Lactobacillus and low abundance of pathogenic bacteria. The balance of bacterial flora was important for pregnancy.
**ENDOMETRIUM**						
Franasiak *et al*., 2016	33	35.9	Ion16S metagenomics V2-4-8, V3-6, V7-9	Pregnancy outcomes of women who underwent single ET of an euploid blastocyst were examined; 18 women were pregnant and 15 were not.	not related	Lactobacillus was the top species call for both outcomes. No differences were found between pregnant and nonpregnant women.
Moreno *et al*., 2016	13	39.5	454 pyrosequencing V3-5	Pregnancy outcomes of women who underwent IVF were compared.	Related	NLDM (<90%) was associated with significant decreases in pregnancy (70.6% *vs*. 33.3%; *p*=.03) and live birth (58.8% *vs*. 6.7%; *p*=.002) rates.
Kyono *et al*., 2019	92	37	Illumina MiSeq V4	Pregnancy outcomes of 92 IVF cases (47 LDM cases and 45 NLDM cases) and 9 NLDM cases receiving antibiotics and prebiotics were compared.	Tend to be related	After intervention, 9 NLDM cases became LDM (46 LDM cases vs. 36 NLDM cases). Pregnancy rates were higher in the LDM group (58.9%) than in the NLDM group (47.2%), but no significant difference was found.
Hashimoto & Kyono, 2019	99	33.5	Illumina MiSeq V4	Pregnancy outcomes of 99 IVF cases were compared between dysbiotic endometrium (n=31) and eubiotic endometrium (n=68).	not related	Pregnancy rates were comparable between eubiotic and dysbiotic microbiome endometria.
Present study	35	36	Illumina MiSeq V1, V2	The endometrial microbiome profiles of 34 women who underwent good ET with IVF were compared (21 pregnant cases vs. 13 nonpregnant cases).	Related	The endometrial microbiomes of pregnant cases demonstrated significantly more cases of high occupancy of Lactobacillus and low occupancy of pathogenic bacteria. The balance of bacterial flora was important for pregnancy.

ART, Assisted reproductive technology; ET, embryo transfer; ICSI, intracytoplasmic sperm injection; IVF, in vitro fertilization; LDM, *Lactobacillus*-dominant microbiota; NLDM, non-*Lactobacillus*-dominant microbiota.

When *Lactobacillus* spp. were examined according to species, CST III (*L. iners* dominant) was the most common in pregnant women, followed by CST II (*L. gasseri* dominant). Contrary to the results of this study, it has been reported that *L. iners* has adverse effects on pregnancy, such as infertility, sexually transmitted diseases, and miscarriage (Novak *et al*., 2022; Chang *et al*., 2020). However, the role of each species of *Lactobacillus* is still unknown. Therefore, further studies are needed to determine whether the effect of *Lactobacillus* on pregnancy differs based on species.

In β-diversity analysis, the vaginal and endometrial microbiota were divided into several similar clusters and examined using the PERMANOVA test. Although high *Gardnerella* abundance was associated with low pregnancy rates, high *Lactobacillus* abundance was associated with high pregnancy rates. These results are similar to those reported by Moreno *et al*. (2016) and are reproducible, supporting the proposed relationship between the endometrial and vaginal microbiomes and pregnancy. Furthermore, *Gardnerella* spp. are highly prevalent in the group of pathological bacteria.

### Mechanisms by which the Reproductive Microbiome Affects Implantation

Generally, the mechanisms by which the endometrial and vaginal microbiomes affect implantation are immunological. Pregnancy depends on the receptive state of the endometrium, which is influenced by hormones, anatomical receptivity, and the immune system (Benner *et al*., 2018). Endometrial and intestinal mucosal immune mechanisms are similar. Based on the results of the well-studied gut microbiota and immune mechanisms, a link between endometrial microbiota and immune mechanisms has been suggested (Benner *et al*., 2018). Gut microbiota regulates T-cell proliferation, macrophage development and function, and neutrophil chemotaxis (Di Simone *et al*., 2020; Chang *et al*., 2014; Thorburn *et al*., 2015). Inflammation in the uterus caused by bacterial infection may also affect cytokines required for blastocyst development and implantation (Sirota *et al*., 2014).

The immune tolerance of some immune cells (e.g., regulatory T cells) may affect implantation (Sasaki *et al*., 2004). Benner *et al*. (2018) suggested that when bacteria invade the endometrium and stimulate the pattern-recognition receptors in epithelial cells, the epithelial cells release cytokines that affect local lymphocyte populations. The endometrial microbiome interacts with host cells in a manner similar to that seen in the mucosal epithelium of the intestine (Benner *et al*., 2018). According to a previous study, the reproductive success rate of germ-free mice after embryo implantation was lower than that of conventionalized mice (Inzunza *et al*., 2005). Thus, the endometrial microbiome plays various roles in implantation (Inzunza *et al*., 2005). These findings indicate that *Lactobacillus* is predominant in the endometrial and vaginal microbiomes because its presence prevents pathological bacteria from entering the uterus by acting on the pattern-recognition receptors of mucosal cells to regulate immune response, such as immune tolerance, necessary for implantation. The endometrial microbiome may regulate immune response, such as the immune tolerance required for implantation. However, the mechanism by which the endometrial microbiome affects implantation remains poorly understood, warranting further research.

This study suggested that the low abundance of *Lactobacillus* spp. and high abundance of pathological bacteria in the vaginal and endometrial microbiomes adversely affect pregnancy. Hence, it is reasonable to speculate that the balance of the pathological bacterial microbiome is important for pregnancy, and intervention is needed for those with Low L + High PB. In future studies, researchers should investigate whether prebiotics, such as lactoferrin, should be administered to increase *Lactobacillus* or whether antibiotics should be administered to decrease pathological bacteria in patients with this type of microbiome. Lactoferrin, an iron-binding cationic glycoprotein, has a complementary relationship with *Lactobacillus* spp., which do not require iron as a nutrient. As a result, it inhibits microbial adhesion to cells and intracellular replication (Valenti *et al*., 2018). However, reports on the efficacy of lactoferrin in increasing endometrial and vaginal *Lactobacillus* spp. and improving pregnancy outcomes are still limited; thus, further research is needed (Corbett *et al*., 2021; Kyono *et al*., 2018a).

The limitation of this study was the small sample size (35 cases only). Nevertheless, the overall quality of the transferred embryos was good in terms of morphology. Due to the situation in Japan, the chromosomes were not examined, and the aneuploidy of the embryos was not unified. Additionally, some cases of chronic endometritis diagnosed using hysteroscopy were treated with antibiotics. Therefore, the same analysis was performed after excluding the cases of antibiotic use in the subanalysis. The results showed a trend, although insignificant, in the endometrial microbiome, and a significant difference in the vaginal microbiome, suggesting an association between pregnancy outcomes and the balance of the endometrial and vaginal microbiomes.

In conclusion, our findings show that the balance between *Lactobacillus* abundance and pathological bacteria abundance in the endometrial and vaginal microbiomes is associated with pregnancy outcome using ART.
